# The clinical results of proton beam therapy in patients with idiopathic pulmonary fibrosis: a single center experience

**DOI:** 10.1186/s13014-016-0637-3

**Published:** 2016-04-18

**Authors:** Takashi Ono, Masato Hareyama, Tatsuya Nakamura, Kanako Kimura, Yuichiro Hayashi, Yusuke Azami, Katsumi Hirose, Yoshiomi Hatayama, Motohisa Suzuki, Hitoshi Wada, Yasuhiro Kikuchi, Kenji Nemoto

**Affiliations:** Department of Radiation Oncology, Southern Tohoku Proton Therapy Center, 7-172, Yatsuyamada, Koriyama, Fukushima 963-8052 Japan; Department of Radiation Oncology, Sapporo Teishinkai Hospital, 1-3-1, Kita33johigashi, Higashi, Sapporo, Hokkaido Japan; Department of Radiation Oncology, Hirosaki University Faculty of Medicine, 5, Zaifu-cho, Hirosaki, Aomori Japan; Department of Radiation Oncology, Yamagata University Faculty of Medicine, 2-2-2, Iida-Nishi, Yamagata, Japan

**Keywords:** Idiopathic pulmonary fibrosis, Idiopathic interstitial pneumonias, Lung neoplasms, Radiation pneumonitis, Protons

## Abstract

**Background:**

The purpose of this study is to retrospectively evaluate the incidence of lung toxicities after proton beam therapy (PBT) in patients with idiopathic pulmonary fibrosis (IPF).

**Methods:**

Patients diagnosed with primary lung cancer or lung metastasis who were treated with PBT between January 2009 and May 2015 were recruited from our database retrospectively. Cases of pneumonitis (excluding infection-related pneumonitis) were evaluated using the Common Terminology Criteria for Adverse Events version 4.0, and the Fletcher-Hugh-Jones classification of respiratory status was used to evaluate pretreatment and posttreatment respiratory function.

**Results:**

Sixteen IPF patients received PBT for lung tumors, 15 received PBT for primary lung cancer, and one patient received PBT for metastasis from lung cancer. The cohort was composed of 14 men and 2 women, with a median age of 76 years (range: 63–89 years). The median follow-up time was 12 months (range: 4–39 months). The median dose of PBT was 80.0 Gy relative biological dose effectiveness (RBE) (range: 66.0–86.4 Gy [RBE]). The cumulative incidence of pneumonitis was 19.8 % (95 % confidence interval [CI]: 0–40.0 %), including one case of grade 5 pneumonitis. Reduced respiratory function was observed after PBT in seven patients, including one patient with pleural dissemination; five of these patients required home oxygen therapy.

**Conclusions:**

This study suggests that PBT can be performed more safely in IPF patients than surgery or X-ray irradiation. Although PBT has become a treatment choice for lung tumors of patients with IPF, the adverse events warrant serious attention.

## Background

Idiopathic interstitial pneumonitis describes a group of diffuse parenchymal lung diseases, the primary injury site of which is the space between the epithelial and endothelial basement membranes. The diseases include idiopathic pulmonary fibrosis (IPF), nonspecific interstitial pneumonia, cryptogenic organizing pneumonia, acute interstitial pneumonia, respiratory bronchiolitis-associated interstitial lung disease, desquamative interstitial pneumonia, and lymphocytic interstitial pneumonia [1].

With an estimated incidence rate of 4.6–16.3 per 100,000, IPF is the most common form of idiopathic interstitial pneumonitis [2]. IPF itself is associated with a poor prognosis; however, IPF patients have a markedly increased risk of lung cancer and IPF is especially problematic for the treatment of lung cancer patients. In IPF patients, the relative risk of lung cancer is reported to be 7.31 [3].

In IPF patients, surgery is complicated by a high rate of respiratory function-related morbidity and a high rate of postoperative mortality [4–6]. Radiotherapy is therefore one of the treatments of choice for patients with IPF who have inoperable lung tumors. However, the performance of chest X-ray irradiation, including conventional radiotherapy and stereotactic body radiotherapy (SBRT) is difficult in IPF patients because of the high rate of life-threatening pneumonitis after treatment [7–9].

At present, an increasing number of lung cancer patients are treated using proton beam therapy (PBT) with or without chemotherapy [10–12]. The advantage of PBT is that it can deliver a more concentrated dose of radiation than conventional radiotherapy or SBRT using X-ray irradiation [13–15]. However, there is little data regarding the use of PBT in the treatment of lung cancer patients who have IPF.

The purpose of the present study is to retrospectively evaluate the incidence of lung toxicities, including pneumonitis and reduced respiratory function after PBT in patients with IPF. The treatment method and procedure were approved by the ethics committees of our institution (Southern TOHOKU Research Institute for Neuroscience) which proceeded according to the Declaration of Helsinki.

## Methods

### Patients

The present study included patients who were diagnosed with primary lung cancer or lung metastasis and who were treated with PBT between January 2009 and May 2015 at the Southern Tohoku Proton Therapy Center. The patients were recruited from our database retrospectively. In the present study, it did not matter whether the pathology of the lung tumor was histologically confirmed or not. The clinical stage of the patients’ lung cancer or lung metastasis was determined using computed tomography (CT) and positron emission tomography (PET)-CT. Written informed consent was obtained from all patients. The inclusion criteria were as follows: a solitary or double lung tumor, a World Health Organization performance status of 0–2, no lymph node metastasis, and the absence of distant organ metastasis except for solitary lung metastasis or other sites of uncontrolled cancer. Patients with solitary lung tumor which seemed lung metastasis were treated for radical radiotherapy dose, because it can’t be clinically judged whether this lung tumor was metastasis or primary lung cancer if the lung tumor can’t be histologically proven.

### Proton beam therapy

**Fig. 1:**
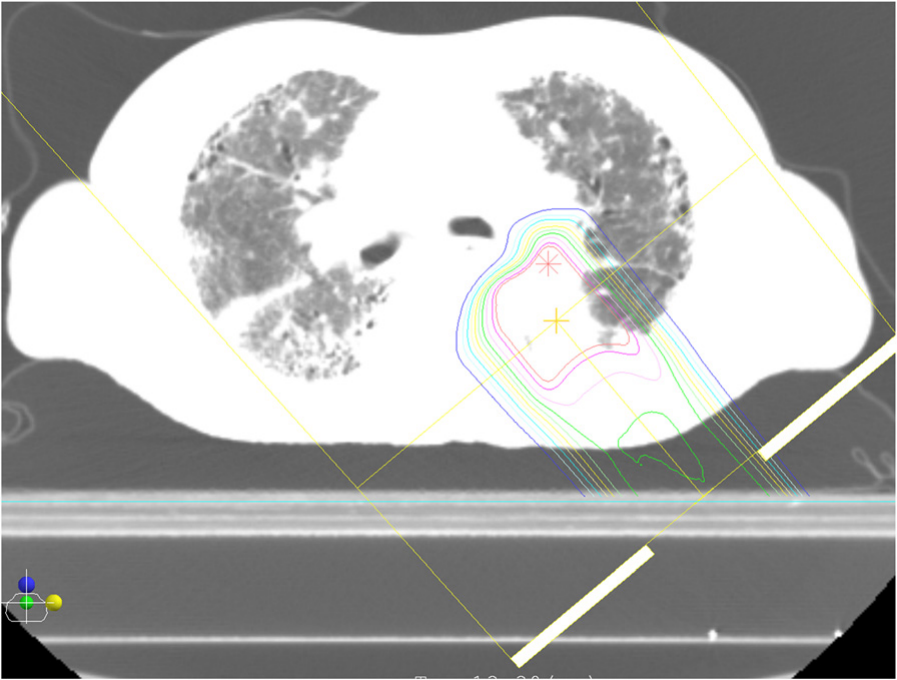
The dose distribution map for proton beam therapy for a lung cancer patient with idiopathic fibrosis. The region outside the outermost line received <10 % radiation

Treatment planning for PBT was based on three-dimensional CT images that were taken at 2 mm intervals in the exhalation phase while using a respiratory gating system (Anzai Medical, Tokyo, Japan). A custom-indexed vacuum-lock bag was used to immobilize the patients. An Xio-M (CMS Japan, Tokyo, Japan; and Mitsubishi Electric) treatment planning system was used to calculate the dose distributions for PBT. The gross tumor volume (GTV) included the lung tumor. The clinical target volume (CTV) was defined as GTV plus 0.5 cm. The planning target volume (PTV) was CTV plus a 0.5 cm margin. When respiratory movement was large, the gap of base position in the exhalation phase was over 30 %, despite the use of the respiratory gating system, a 0.2–0.5 longitudinal margin was added to the PTV. Proton energy levels of 150 MeV and 210 for 2–3 fields, and a spread-out Bragg peak were tuned to the extent that was possible until the PTV was exposed to a 90 % isodose of the prescribed dose (Fig. 1). The PBT system at our institute (Proton Beam System, Mitsubishi, Tokyo, Japan) used a synchrotron, and a passive scattering method in which a proton beam passes a bar ridge filter, a range shifter, and a customized compensator before entering the patient. Treatment was administered during the exhalation phase using a respiratory gating system. A multileaf collimator, which consisted of 40 iron plates with a width of 3.75 mm, and which could be formed into an irregular shape, was used. Daily front and lateral X-ray imaging was used for positioning. The PBT schedule was 66 Gy relative biological dose effectiveness (RBE) in 10 fractions over 2 weeks for peripheral lung tumors, and 80 Gy (RBE) in 25 fractions over 5 weeks for central or centrally located lung tumors. The biologically equivalent dose of 10 for 66 Gy (RBE) and 80 Gy (RBE) was 109.56 Gy (RBE) and 105.6 Gy (RBE) respectively. Patients with lung tumors located near the large intestine or small intestine and those with severe respiratory dysfunction (such as patients who required home oxygen therapy), received 79.2–86.4 Gy (RBE) in 33–36 fractions over 7 weeks. Dose constrains were set for the esophagus (≤55 Gy [RBE]), spinal cord (≤40 Gy [RBE]), trachea/bronchus (≤55 Gy [RBE]), and heart (≤40 Gy [RBE]).

### Evaluation and follow-up

All patients underwent either CT or PET-CT to evaluate the initial tumor response within 3 months of the completion of treatment. The follow-up interval was every 1–3 months for the first year and every 3–6 months thereafter. IPF was diagnosed with the use of high resolution CT. The radiographic diagnosis of IPF was based on a bilateral, predominantly basal, predominantly subpleural, reticular pattern associated with sub pleural cysts [1]. Pneumonitis, excluding infection, was evaluated using the Common Terminology Criteria for Adverse Events version 4.0 [16]. Infectious pneumonitis was defined pneumonitis which proven bacterial infection by germ culture, and patients recovered by antibiotic. The Fletcher-Hugh-Jones classification of respiratory status was used for evaluating pretreatment and posttreatment respiratory function (class 1, the patient’s breathing is similar to others of the same sex and age; class 5, the patient is breathless when talking or undressing, or is unable to leave the house due to breathlessness) [17]. The following dosimetric factors were examined with the use of a dose volume histogram of the lung minus the GTV: mean lung dose (MLD), lung V5, lung V10, lung V15, lung V20, lung V25, and lung V30.

### Statistical analysis

All statistical analyses were performed using the IBM SPSS Statistics version 22 software package (SPSS Inc., Chicago, IL, USA). The overall survival (OS) time was defined as the time between the start of treatment and the last follow-up. The Kaplan-Meier method was applied to estimate survival probability. Kaplan-Meier algorithms was applied to estimate the cumulative incidence of pneumonitis. The relationships between the occurrence of pneumonitis and the dose volume histogram factors were examined using the Mann–Whitney *U* test. All *p*-values were two sided, and *p* <0.05 were considered to indicate statistically significant.

## Results

### Patients

There were 23 patients with interstitial pneumonitis received PBT, however four patients did not fulfill IPF criteria. Nineteen IPF patients received PBT for a lung tumor. Of these 19 patients, two were excluded from the analysis due to lymph node metastasis; one was excluded due to distant other organ metastasis. The characteristics of 16 patients, including 14 patients with inoperable cancer, are summarized in Table 1. Four out of the 16 patients received treatment for IPF (steroid administration [*n* = 2], and home oxygen therapy [*n* = 2]) before receiving PBT for the lung tumor. Two patients underwent sequential treatment for two lung tumors at different sites. Fifteen patients received PBT for primary lung cancer, and one patient received PBT for metastasis from lung cancer. The cohort was composed of 14 men and two women, with a median age of 76 years (range: 63–89 years). The median follow-up time was 14 months (range: 5–39 months). Ten Patients were histologically proven (seven were squamous cell carcinoma, two were adenocarcinoma, and one was small cell carcinoma). The median dose of PBT was 80.0 Gy (RBE) (range: 66.0–86.4 Gy [RBE]).

**Table 1:** The patient characteristics

Characteristics	Patients
Age (years)	
Median (range)	76 (63–89)
Gender	
Male	14 (87 %)
Female	2 (13 %)
Performance status	
0	9 (56 %)
1	5 (31 %)
2	2 (13 %)
Follow-up time (months)	
Median (range)	14 (5–39)
T category^a^ (*n* = 15)	
T1	7 (47 %)
T2	6 (40 %)
T3	2 (13 %)
Stage^a^ (*n* = 15)	
I	12 (80 %)
II	3 (20 %)
Histopathology	
Squamous cell carcinoma	7 (44 %)
Adenocarcinoma	2 (13 %)
Small cell carcinoma	1 (6 %)
Clinical malignancy	5 (31 %)
Metastasis (lung cancer)	1 (6 %)
Diameter of lung tumor (mm)	
Median (range)	24 (15–60)
Total dose (Gy (RBE))	
Median (range)	80 (66.0–86.4)

*Abbreviations: RBE* relative biological dose effectiveness, *PBT* proton beam therapy

^a^Numbers correspond to the tumor-node-metastasis system of classification (International Union Against Cancer criteria)

### Pneumonitis

**Fig. 2:**
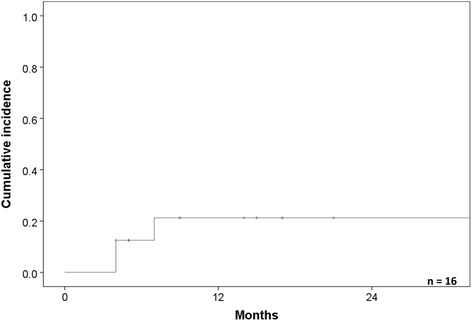
The cumulative incidence of radiation pneumonitis. The cumulative incidence was 19.8  %

The cumulative incidence of pneumonitis was 19.8 % (95 % confidence interval [CI]: 0–40.0 %) (Fig. 2), including one case of grade 5 pneumonitis (Table 2). Two cases (one was grade 2 pneumonitis, and another one was grade three pneumonitis) of pneumonitis were occurred within 4 months, and one case occurred within 7 months. A patient who developed grade 5 pneumonitis underwent PBT for squamous cell carcinoma of the left lower lobe. The tumor was inoperable due to poor respiratory function and IPF. The patient received 80 Gy (RBE) in 25 fractions. Three months after the first PBT treatment, the patient received 59.4 Gy (RBE) in nine fractions for a lung tumor of the right upper lobe, adjacent to the pleura. Three months after the second treatment, the patient experienced dyspnea due to radiation pneumonitis. The patient was treated with steroids and the dyspnea was relieved; however, the patient died due to acute respiratory failure 1 month later. There were no statistically significant differences with regard to the dosimetric factors in relation to the lung and the occurrence of grade 3–5 pneumonitis (Table 3).

**Table 2:** The grade of pneumonitis and respiratory function deterioration

	Age	Grade of pneumonitis	Pre FHJ	Post FHJ	MLD	Lung V20	Operable/inoperable	IPF treatment before PBT	IPF treatment after PBT	Deterioration of IPF shadow
1	69	1	1	3	8.1	14.8	Inoperable	-	HOT	+
2	77	1	2	2	3.7	5.7	Operable	-	-	-
3	85	1	1	2	5.6	9.1	Inoperable	-	-	-
4	66	1	2	3	6.3	10.4	Inoperable	-	-	+
5	78	3	3	4	4.2	6.9	Inoperable	-	HOT	+
6	71	2	2	2	3.0	4.7	Inoperable	Steroid	Steroid	-
7	75	1	1	1	9.4	16.1	Inoperable	-	-	+
8	83	1	1	1	0.9	4.2	Inoperable	-	-	-
9	89	5	1	3	10.6	17.9	Inoperable	-	HOT	+
10	63	1	1	1	6.8	12.8	Inoperable	Steroid	Steroid	+
11	80	1	1	3	4.2	6.9	Inoperable	-	HOT	+
12	69	1	1	3	3.8	6.3	Inoperable	-	HOT	-
13	72	1	4	4	1.8	2.9	Inoperable	HOT	HOT	-
14	75	1	2	2	8.7	14.6	Inoperable	-	-	-
15	77	1	1	1	4.7	8.5	Operable	-	-	-
16	79	1	4	4	3.4	5.5	Inoperable	HOT	HOT	-

*Abbreviations: Pre FHJ* Fletcher-Hugh-Jones classification before proton beam therapy, *Post FHJ* Fletcher-Hugh-Jones classification after proton beam therapy, *MLD* mean lung dose, *IPF* idiopathic pulmonary fibrosis, *HOT* home oxygen therapy

**Table 3:** The relationship between the dose volume histogram parameters and the incidence of grade 3–5 pneumonitis

	Grade ≥3 pneumonitis (*n* = 2)	Grade ≤2 pneumonitis (*n* = 14)	*p* value
	mean ± SD	mean ± SD	
MLD	7.4 ± 4.5	5.0 ± 2.6	0.333
Lung V5	16.5 ± 9.3	12.3 ± 5.3	0.417
Lung V10	14.6 ± 8.8	10.8 ± 4.9	0.500
Lung V15	13.4 ± 8.2	9.7 ± 4.6	0.417
Lung V20	12.4 ± 7.8	8.8 ± 4.3	0.333
Lung V25	11.6 ± 7.3	8.0 ± 4.0	0.333
Lung V30	10.8 ± 6.9	7.3 ± 3.8	0.333

*Abbreviations: MLD* mean lung dose, *SD* standard deviation

### Respiratory function deterioration

Seven patients showed deterioration in their respiratory status (based on the Fletcher-Hugh-Jones classification of respiratory status) after PBT (Table 2). One of the seven patients had a high volume of pleural effusion due to pleural dissemination. Five of the seven patients were newly introduced home oxygen therapy. In two of the five patients, deterioration of the pneumonitis shadow was observed at the site of the PBT treatment, for which they required treatment. Exacerbation occurred in four patients within 6 months, one patient within 1 year, and two patients within 2 years. Deterioration of the IPF shadow on high resolution CT was observed in two patients, five of whom experienced exacerbation.

### Survival

**Fig. 3:**
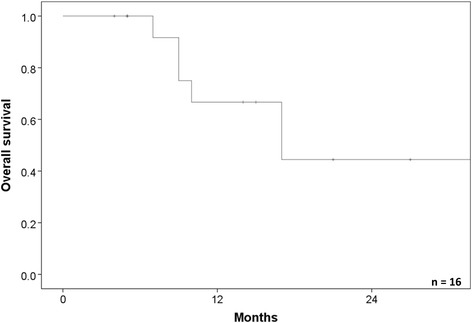
The overall survival rate of the patients who were treated lung cancer or lung metastasis with interstitial pneumonitis. The 1- and 2-year overall survival rates were 69.6 and 44.4 %, respectively

The 1-year and 2-year overall survival rates were 69.6 % (95 % CI: 44.7–94.5 %) and 44.4 % (95 % CI: 15.2–77.6 %), respectively (Fig. 3). Four patients died due to lung cancer (metastasis [*n* = 3]; local recurrence [*n* = 1]), three died due to other causes, and one died due to radiation pneumonitis.

## Discussion

The present study revealed there were 20 % rate of pneumonitis, including one case of grade 5 pneumonitis and high rate of respiratory function deterioration, even when PBT was used to treat the lung tumors of IPF patients. To the best of our knowledge, this is the first report on the incidence of toxicity after PBT in IPF patients.

Radiation pneumonitis occurred when patients received radiotherapy for lung cancer. Grade ≥3 pneumonitis occurred at a rate of 1.3–20 % [18–21] in patients who underwent SBRT and 1.4–17 % [22, 23] in patients who underwent radiotherapy with or without chemotherapy for stage 3 lung cancer. On the other hand, in the lung cancer patients who underwent PBT, pneumonitis occurred at a rate of 0–8 % [10–12]. The high incidence (20 %) of grade ≥3 pneumonitis reported by Yamashita et al. [19], may have occurred because two of the five patients with grade ≥3 suffered from IPF. In contrast, 13 % patients of the patients had grade ≥3 pneumonitis in the present study. This rate was relatively high, and suggests that the occurrence of pneumonitis was increased even though the IPF patients were treated with PBT.

Table 4 summarizes the rate of grade 4 or 5 pneumonitis in IPF patients in previous studies and the present study [7–9]. These studies reported that the rate of occurrence was 19–53.8 %. Yamashita et al. [8] and Lee et al. [9] reported that IPF increased the risk of radiation pneumonitis. Although Yamaguchi et al. [7] reported that IPF was not a significant factor for grade ≥2 radiation pneumonitis, they reported that radiation pneumonitis frequently occurred in IPF patients. These studies suggest that IPF increases the risk of life-threatening pneumonitis after X-ray irradiation. In fact, radiation pneumonitis occurred at a higher rate than in patients without IPF who underwent X-ray irradiation [18, 20–23]. In the present study grade 4 or 5 pneumonitis occurred at a rate of 6.3 %, which is lower than that reported in previous studies. This suggests that PBT reduced the incidence of life-threatening pneumonitis in IPF patients who were treated lung tumor in comparison to IPF patients who were treated with X-ray irradiation (including conventional radiotherapy and SBRT). This may be because PBT can deliver a smaller lung dose-volume than X-ray irradiation [13–15]. This suggests that PBT can become a treatment choice for lung cancer patients with IPF. There were no statistically significant differences in the dosimetric factors of the patients, and there were two patients in the present study who developed pneumonitis despite the low values of their MLD, lung V5, lung V10, lung V15, lung V20, lung V25, and lung V30. However, it should be noted that Yamaguchi et al. [7] and Lee et al. [9] reported a correlation between the dosimetric factors and the occurrence of pneumonitis in a study that included IPF patients. Thus, the risk of pneumonitis after radiotherapy may increase in IPF patients, even though the radiation fields are small.

**Table 4:** The previous studies on grade 4 or 5 pneumonitis after radiation therapy in patients with interstitial pneumonitis

	Number of patients	Treatment	Median total dose	Pneumonitis
Yamaguchi et al. [7]	16	SBRT	48	3 (19 %)
Yamashita et al. [8]	13	SBRT	48	7 (53.8 %)
Lee et al. [9]	15	Conformal RT	56.9	5 (33.3 %)
Present study	16	PBT	80 Gy (RBE)	1 (6.3 %)

*Abbreviations: SBRT* stereotactic body radiotherapy, *RT* radiotherapy, *RBE* relative biological dose effectiveness, *PBT* proton beam therapy

IPF is a disease that is associated with a poor prognosis. The median survival of patients with IPF is 2–3 years from the time of diagnosis [2]. Moreover, IPF may be acutely exacerbated by a range of factors, including infection and surgery [24]. Nagato et al. [25] and Takeda et al. [26] reported one case each of IPF exacerbation after PBT and SBRT, respectively. Respiratory function deterioration (except for that which occurred due to cancer progression) was observed in 38 % of the patients of the present study. In the present study we did not determine whether this was an exacerbation of IPF or a comorbidity after PBT because all of the patients showed IPF shadow deterioration on CT. However, it seemed that a relatively high number of patients (five of 14 patients [35.7 %]) acquired the need for home oxygen therapy after undergoing PBT for lung tumors without lymph node metastasis. This suggests that not only X-ray irradiation but also PBT may be a trigger for the exacerbation of IPF.

Surgery including lobectomy and biopsy also exacerbate IPF. The rate of postoperative IPF exacerbation is reported to be 9.3–30 % [4–6]. In the present study, with the exception of one patient who experienced exacerbation due to lung cancer progression, six patients (37 %) had exacerbation. The rate of exacerbation in the present study was higher than the rate attributed to surgery. This may be because the previous reports observed acute exacerbation within 30 days and because there the present study included patients with inoperable tumors. In the present study, no patients experienced acute exacerbation within 30 days. In that sense, PBT may be safer option for the treatment of lung cancer patients with IPF than surgery.

## Conclusions

The present study may indicate that PBT is a safer procedure for the treatment of lung cancer in IPF patients than surgery, SBRT, and conventional radiotherapy. Although PBT may become a treatment choice for lung cancer patients with IPF, we should pay close attention to the occurrence of adverse events, including pneumonitis and the exacerbation of IPF after PBT.

## Abbreviations

CT: computed tomography
CTV: clinical target volume
GTV: gross tumor volume
IPF: idiopathic pulmonary fibrosis
MLD: mean lung dose
OS: overall survival
PBT: proton beam therapy
PET: positron emission tomography
PTV: planning target volume
RBE: relative biological dose effectiveness
SBRT: stereotactic body radiotherapy
